# Selenium‐Doped Carbon Quantum Dots Act as Broad‐Spectrum Antioxidants for Acute Kidney Injury Management

**DOI:** 10.1002/advs.202000420

**Published:** 2020-04-29

**Authors:** Zachary T. Rosenkrans, Tuanwei Sun, Dawei Jiang, Weiyu Chen, Todd E. Barnhart, Ziyi Zhang, Carolina A. Ferreira, Xudong Wang, Jonathan W. Engle, Peng Huang, Weibo Cai

**Affiliations:** ^1^ Department of Pharmaceutical Sciences University of Wisconsin‐Madison Madison WI 53705 USA; ^2^ Marshall Laboratory of Biomedical Engineering International Cancer Center Laboratory of Evolutionary Theranostics School of Biomedical Engineering Shenzhen University Health Science Center Shenzhen 518060 China; ^3^ Departments of Radiology and Medical Physics University of Wisconsin‐Madison Madison WI 53705 USA; ^4^ Department of Materials Science and Engineering University of Wisconsin‐Madison Madison WI 53705 USA

**Keywords:** acute kidney injury, carbon quantum dots, cisplatin, nanomedicine, positron emission tomography, rhabdomyolysis, selenium

## Abstract

The manifestation of acute kidney injury (AKI) is associated with poor patient outcomes, with treatment options limited to hydration or renal replacement therapies. The onset of AKI is often associated with a surfeit of reactive oxygen species. Here, it is shown that selenium‐doped carbon quantum dots (SeCQDs) have broad‐spectrum antioxidant properties and prominent renal accumulation in both healthy and AKI mice. Due to these properties, SeCQDs treat or prevent two clinically relevant cases of AKI induced in murine models by either rhabdomyolysis or cisplatin using only 1 or 50 µg per mouse, respectively. The attenuation of AKI in both models is confirmed by blood serum measurements, kidney tissue staining, and relevant biomarkers. The therapeutic efficacy of SeCQDs exceeds amifostine, a drug approved by the Food and Drug Administration that also acts by scavenging free radicals. The findings indicate that SeCQDs show great potential as a treatment option for AKI and possibly other ROS‐related diseases.

## Graphical abstract

The specific renal accumulation and excellent antioxidant properties of SeCQDs allows prevention and treatment of acute kidney injury.

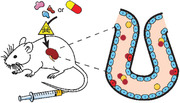

## 1 Introduction

Acute kidney injury (AKI) often complicates treatment outcomes of hospitalized patients, which manifests as decreased kidney glomerular filtration and an increased accumulation of nitrogen waste products in the blood.^[^ ^1^ ^]^ These complications lead to high morbidity and mortality, with an estimated incidence of one in five hospitalized adult patients.^[^ ^2^ ^]^ Currently, AKI treatment predominately relies on supportive therapies such as hydration or renal replacement therapies. Clinicians often encounter two forms of AKI, such as rhabdomyolysis‐induced AKI (RM‐AKI) or cisplatin‐induced AKI (CP‐AKI), although other instances exist.^[^ ^3^ ^]^ RM‐AKI commonly occurs after the breakdown and necrosis of skeletal muscle releases pernicious proteins, largely myoglobin, and electrolytes into circulation.^[^ ^4^ ^]^ CP‐AKI frequently arises from the nephrotoxicity associated with the use of cisplatin in cancer patients, which occurs in about 1/3 of all patients.^[^ ^5^ ^]^ Oxidative stress leading to acute tubular necrosis from reactive oxygen species (ROS) has been implicated in the pathogenesis of both conditions, as is the case for many cases of AKI.^[^ ^6^ ^]^ Amifostine (AMF) is a drug approved by the U.S. Food and Drug Administration (FDA) to prevent cisplatin‐induced toxicity with a mechanism of free radical scavenging.^[^ ^7^ ^]^ Side‐effects and limited efficacy of AMF have hampered its clinical utilization, but the successful development provides a foundation for future therapies.

Nanotechnology continues to transform medicine for disease diagnosis and treatment. The ideal fabrication of nanomaterials is generally believed to permit controlled and optimized delivery to diseased tissues in vivo for disease diagnosis and therapy, collectively called theranostics.^[^ ^8^, ^9^ ^]^ However, the application of nanotechnology for renal disease management is still in its infancy, but the prospects are promising.^[^ ^10^ ^]^ Recent efforts have made significant progress in theranostic nanosystems for the clinical diagnosis and treatment of renal diseases.^[^ ^11^ ^]^ Nanomaterials can overcome the limitations of the current clinical gold standards for the detection of renal diseases, such as glomerular filtration rate, by assessing the state of kidneys non‐invasively, in real‐time, and with excellent signal‐to‐background ratios.^[^ ^12^ ^]^ Additionally, with proper design, nanosystems can treat renal disease with efficacy superior to small‐molecule drugs.^[^ ^13^ ^]^ These studies, along with others, have highlighted the nanomaterial properties required to reach and filter through the urinary system and treat renal diseases.^[^ ^14^, ^15^ ^]^ Carbon quantum dots (CQDs) have emerged as attractive nanomaterials due to ease of synthesis, high biocompatibility, low cost, and tunable fluorescent properties.^[^ ^16^ ^]^ These advantages have encouraged the replacement of semiconductor quantum dots with CQDs in biosensing, imaging, and therapeutic applications.^[^ ^17^ ^]^ In comparison to the limited bioavailability of small molecules, CQDs can be designed to maximize kidney accumulation to most effectively treat renal diseases. Doping CQDs with elements has been shown to engineer them with intrinsic properties effectively.^[^ ^18^ ^]^ An example is selenium, an essential trace element, which has a vital role as an antioxidant in many physiological processes through incorporation into selenoproteins.^[^ ^19^ ^]^ Designing nanomaterials with antioxidant properties using this method is in accordance with ongoing research that shows they are superior to small molecules due to their controlled biodistribution and intrinsic scavenging abilities.^[^ ^20^ ^]^

**Figure 1:**
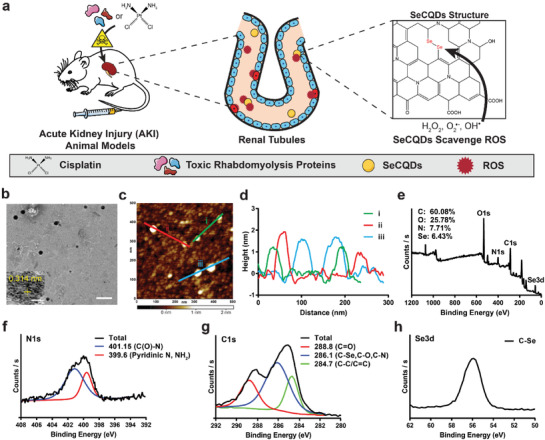
a) Scheme showing the specific renal accumulation of SeCQDs allows prevention and treatment of AKI of different origins. b) TEM image of SeCQDs. Scale bar: 200 nm; Inset: High‐resolution TEM image of SeCQDs. Scale bar: 10 nm. c) AFM image. d) corresponding height profile analysis. e) XPS survey and high‐resolution spectra of f) C1s, g) N1s and h) Se3d.

In this study, selenium‐doped carbon quantum dots (SeCQDs) were synthesized using a simple hydrothermal treatment. In vitro experiments ascertained that SeCQDs were potent ROS scavengers. We then utilized positron emission tomography (PET) imaging to elucidate the high kidney uptake of SeCQDs in vivo using Zr‐89 (*t*_1/2_ = 78.4 h). SeCQDs were shown to be robust therapies to treat (RM‐AKI) and prevent (CP‐AKI) AKI due to the high renal accumulation and excellent antioxidant properties (**Figure** **1**a). Comparison to the FDA approved small molecule, AMF, further demonstrated the superiority of this nanomaterial to small molecules for AKI treatment. These results will continue to inspire the development of therapeutic applications for nanomedicine in ROS‐related and renal diseases using AKI as a model.

## 2 Results and Discussion

Selenium‐doped carbon quantum dots (SeCQDs) were prepared using a hydrothermal method.^[^ ^21^ ^]^ Briefly, l‐selenocysteine was dispersed in alkaline conditions and formed water‐soluble SeCQDs after 24 h at 60 °C (Figure S1, Supporting Information). Morphological characterization of SeCQDs was performed using transmission electron microscopy (TEM) and atomic force microscopy (AFM). These studies show that SeCQDs have a diameter and height of approximately 40 and 2 nm, respectively (Figure 1b–d). The structure of the SeCQDs was further analyzed using X‐ray photoelectron spectroscopy (XPS), which revealed SeCQDs were primarily composed of carbon, oxygen, nitrogen, and selenium (Figure 1e–h). High‐resolution XPS spectra were used to determine the atomic compositions, which show a higher selenium content (6.43%) than previous reports.^[^ ^21^ ^]^ Furthermore, the high‐resolution XPS spectra were used to ascertain the chemical states of each element by peak deconvolution. Zeta potential measurements determined that the surface was highly negatively charged (−31.0 ± 1.4 mV), attributed to surface carboxyl and hydroxyl groups (Supplementary Information, Figure S2a). X‐ray diffraction found a broad peak at about 22°, indicative of an amorphous structure and (002) interlayer spacing of 0.34 nm that is similar to bulk graphite (Figure S3, Supporting Information).^[^ ^22^ ^]^ UV–vis analysis found peaks at approximately 280, 340, and 470 nm due to the presence of multiple electron transitions (Figure S4, Supporting Information).^[^ ^23^ ^]^ The fluorescence spectra showed primary excitation and emission peaks at 396 and 496 nm, respectively (Figure S5, Supporting Information).

**Figure 2:**
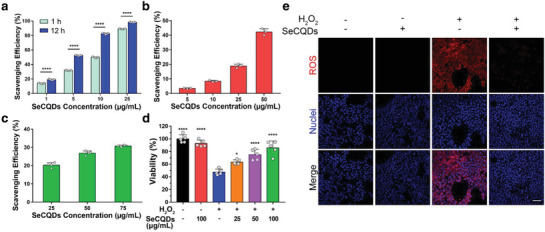
Broad‐spectrum antioxidant activities of SeCQDs in vitro. ROS scavenging efficiencies were evaluated for a) ABTS free radicals, b) ^●^OH, and c) O_2_^●−^. d) Cell viability assay. e) Confocal imaging of ROS production using CellROX Deep Red Reagent in HEK293 cells treated with 250 µm H_2_O_2_. Scale bar: 100 µm. Data represents means ± s.d. from a) five, b) three, or c,d) four independent replicates. *p* values were calculated using a) two‐tailed Student's *t*‐test or d) one‐way ANOVA with Tukey's honest significant different post‐hoc test (*p < 0.05, ****p < 0.0001).

With selenium successfully incorporated into the carbon quantum dots, we then evaluated their capacity to scavenge various free radicals. The antioxidant properties of SeCQDs were first investigated using 2,2'‐azino‐bis(3‐ethylbenzothiazoline‐6‐sulfonic acid) (ABTS) free radicals. As shown in **Figure** **2**a, incubating SeCQDs with ABTS free radicals demonstrates SeCQDs efficiently scavenge these radicals in a concentration and time‐dependent manner. In additional assays, physiologically relevant ROS, superoxide anion radicals (O_2_^●−^), and hydroxyl radicals (OH^●^) further show excellent ROS scavenging abilities of SeCQDs (Figure 2b,c). The mechanism of free radical scavenging was investigated using XPS after incubating SeCQDs with hydrogen peroxide (H_2_O_2_), another ROS that is known to cause oxidative stress in diseases such as AKI. XPS results showed that H_2_O_2_ induced a change only in the selenium spectra, attributed to the formation of selenic acid (Figure S6, Supporting Information).

The kidney is vulnerable to damage from ROS because it receives approximately 25% of the blood supply.^[^ ^24^ ^]^ The imbalance of ROS, referred to as oxidative stress, is detrimental to renal tubules and ultimately leads to AKI.^[^ ^25^ ^]^ As such, we investigated whether SeCQDs were able to protect human embryonic kidney 293 (HEK293) cells from oxidative stress. First, we found that SeCQDs exhibit no significant cytotoxicity using an MTT assay (Figure S7, Supporting Information). To evaluate whether SeCQDs were able to protect HEK293 cells, we induced oxidative stress with H_2_O_2_ (Figure 2d). HEK293 cells were cultured with 250 µm H_2_O_2_, which decreased cell viability by approximately 50%. However, cell viability drastically increased after adding SeCQDs to the culture medium. The protective effects of SeCQDs were attributed to decreased intracellular ROS production, as shown in Figure 2e. Lastly, SeCQDs reduced mitochondrial dysfunction when oxidative stress from H_2_O_2_ was induced in HEK293 cells (Figure S8, Supporting Information).

**Figure 3:**
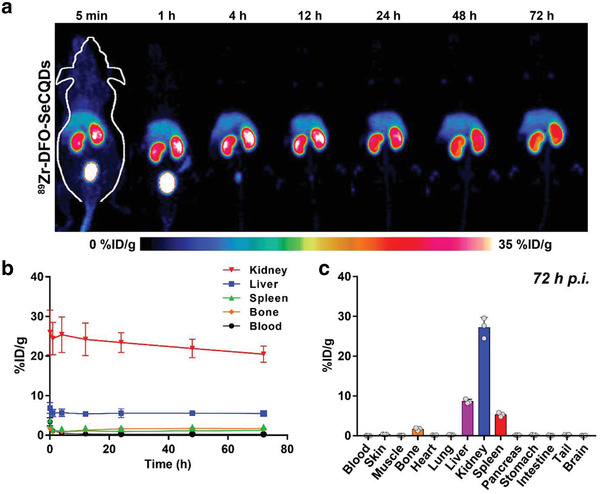
Biodistribution of SeCQDs in healthy mice. a) MIP PET images for ^89^Zr‐DFO‐SeCQDs in healthy ICR mice and b) corresponding ROI analysis. c) Ex vivo biodistribution. In (b,c), data represent means ± s.d. from three independent replicates.

Longitudinal PET imaging studies were performed to investigate the biodistribution of SeCQDs in healthy ICR mice (**Figure** **3**a). To do so, SeCQDs were conjugated with the chelator deferoxamine (DFO), which was confirmed from the C = O stretching vibration (1615 cm^−1^) and N—H stretching and C—N bending vibrations in the FT‐IR spectra (Figure S9, Supporting Information). The zeta potential and diameter of DFO‐SeCQDs were −20.83 ± 0.72 mV and diameter of 52 ± 13 nm, comparable to SeCQDs (Figures S2b and S10, Supporting Information). The successful conjugation of DFO was also indirectly shown by radiolabeling SeCQDs and DFO‐SeCQDs with Zr‐89 and eluting them through a PD‐10 column (Figure S11, Supporting Information). DFO‐SeCQDs were then radiolabeled with Zr‐89 to generate ^89^Zr‐DFO‐SeCQDs for subsequent PET imaging studies. The radiolabeling efficiency determined using thin‐layer chromatography was 84.9 ± 2.0% (*n* = 3; Figure S12, Supporting Information). The radiolabeling stability of ^89^Zr‐DFO‐SeCQDs was then evaluated in 50% FBS and PBS with an EDTA challenge at 37 °C for up to 72 h. In both cases, high radiolabeling stability (>80%) was observed at 72 h (Figure S13, Supporting Information). ^89^Zr‐DFO‐SeCQDs were then intravenously administered into healthy mice, and their biodistribution was monitored up to 72 h using PET imaging. The PET images show that SeCQDs almost exclusively accumulated in the kidneys. The renal uptake of SeCQDs was rapid, as the kidneys were clearly observed at 5 min post‐injection (p.i.). SeCQDs were then slowly eliminated from kidneys up to the 72 h period of the study.

Region‐of‐interest (ROI) analysis of PET images was used to quantify SeCQDs uptake in the major organs (blood, liver, spleen, kidney, bone) (Figure 3b). The kidney uptake reached a maximum of 25.4 ± 4.5% injected dose per gram (%ID/g) (*n* = 3). In comparison, the nonspecific uptake of SeCQDs in the reticuloendothelial system (RES) was drastically lower, with liver and spleen uptake of approximately 5.5 %ID/g and 2.0 %ID/g at 72 h, respectively (*n* = 3). To evaluate the overall elimination of SeCQDs, we quantified the percent injected dose remaining in the mice (Figure S14a, Supporting Information). We found that SeCQDs are eliminated rapidly at approximately 4 h p.i. and then continued to be slowly eliminated up to 72 h (38.2 ± 2.1 %ID; *n* = 3).

Ex vivo biodistribution studies were used to validate the ROI analysis (Figure 3c). Following the last PET scan at 72 h, the mice were euthanized, and the organs of interest were harvested. Similar to the previous analysis, the most significant accumulation was in the kidneys, with uptake of 27.2 ± 2.6 %ID/g (*n* = 3). In comparison, the uptake in the liver and spleen was only 8.6 ± 0.5 and 5.3 ± 0.5 %ID/g, respectively (*n* = 3). Other than bone, which absorbs free Zr‐89, the remaining organ uptake values were less than 0.5 %ID/g.

The relatively low uptake of SeCQDs in the RES organs could be attributed to several factors. As the SeCQDs are forming, their surface is passivated with various functional groups carboxyl, amine, etc.^[^ ^26^ ^]^ This passivated surface might enable SeCQDs to pass through the liver stealthily without significant uptake by macrophages. Therefore, SeCQDs possess intrinsic antifouling properties and limit protein corona formation in systemic circulation. In addition, negatively charged nanoparticles have been shown to further decrease RES uptake by limiting protein corona formation and decreasing interaction with phagocytic cells.^[^ ^9^, ^27^ ^]^

The anatomic barriers that impede the renal clearance of nanoparticles are referred to collectively as the glomerular filtration barrier (GFB). To be filtered thought the GFB, nanoparticles must circumvent glomerular endothelial cells fenestrate (70–90 nm), glomerular basement membrane pores (2–8 nm), and podocyte slits (4–11 nm).^[^ ^28^ ^]^ However, exceptions to size‐scaling laws of renal clearance have been well documented.^[^ ^29^ ^]^ The thickness (<2 nm) of large diameter (1 µm) graphene oxide sheets was found to be a critical factor for urinary excretion.^[^ ^30^ ^]^ Similarly, previous studies showed that glomerular filtration of carbon nanotubes (200–300 nm in length and 20–30 nm in diameter) is possible by perpendicular alignment with the GFB.^[^ ^31^ ^]^ In an analogous process, the rapid renal filtration of SeCQDs is likely facilitated by the thin shape that permits them to pass through the barrier and into the urine after aligning appropriately. Renal accumulation was observed for large, negatively charged gold nanoparticles and DNA origami nanostructures in the renal corpuscle or peritubular capillaries.^[^ ^15^, ^32^ ^]^ In another study, poly(lactic‐co‐glycolic acid) and polyethylene glycol (PLGA‐PEG) nanoparticles were found to selectively accumulate in proximal tubule epithelial cells after transcytosis of peritubular epithelial cells.^[^ ^33^ ^]^ Only through the power of PET imaging were we able to visualize the favorable renal accumulation and quantify it in real‐time using living subjects. With the promising results, we continued to investigate SeCQDs using two AKI animal models.

**Figure 4:**
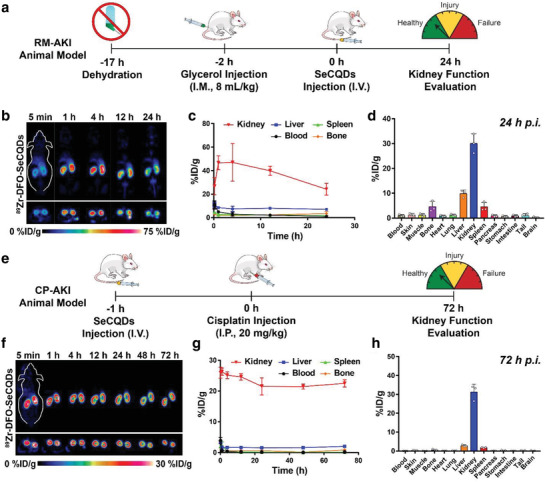
Schemes of AKI animal models and corresponding biodistribution studies using ^89^Zr‐DFO‐SeCQDs. a) Method used to establish RM‐AKI. b) Coronal and axial slices of PET images after administering ^89^Zr‐DFO‐SeCQDs in RM‐AKI model and c) corresponding ROI analysis. d) Ex vivo biodistribution studies in RM‐AKI mice. e) Method used to establish CP‐AKI. f) Coronal and axial slices of PET images after administering ^89^Zr‐DFO‐SeCQDs in CP‐AKI model and g) corresponding ROI analysis. h) Ex vivo biodistribution studies in CP‐AKI mice. In (c–e,g), data represent mean ± s.d. from three independent replicates.

The biodistribution of SeCQDs in the AKI models of interest, RM‐AKI and CP‐AKI, was then investigated using PET imaging studies. RM‐AKI was induced by injecting 50% glycerol intramuscularly into both hindlimbs of the mice after a dehydration period (**Figure** **4**a). After 2 h, increases in blood urea nitrogen (BUN) and creatinine (CRE), as well as tubular damage, are observable.^[^ ^15^, ^34^ ^] 89^Zr‐DFO‐SeCQDs were then intravenously injected. The renal accumulation was again rapid and the most prominent (Figure 4b). The renal uptake of ^89^Zr‐DFO‐SeCQDs in RM‐AKI mice was much higher than in healthy mice (Figure S15, Supporting Information). This difference is attributed to the decreased glomerular filtration resulting from damaged kidneys associated with AKI in RM‐AKI mice.^[^ ^35^ ^]^ ROI analysis indicated a maximum kidney uptake of 47.0 ± 16.0 %ID/g (*n* = 3) at 4 h p.i. (Figure 4c). Elimination of SeCQDs from the kidneys was observed in the remaining time points (Figure S14b, Supporting Information). Ex vivo biodistribution studies at 24 h p.i confirmed the high kidney uptake, which was 30.0 ± 3.9 %ID/g (*n* = 3; Figure 4d).

CP‐AKI was induced by intraperitoneally injecting a large dose of cisplatin (20 mg kg^−1^; Figure 4e). Since clinicians can schedule cancer treatment regimens employing cisplatin, ^89^Zr‐DFO‐SeCQDs were intravenously injected 1 h before administering cisplatin. SeCQDs quickly and almost exclusively accumulated in the kidneys (Figure 4f) as was observed in healthy mice. The kidney uptake reached a maximum of 26.3 ± 1.2 %ID/g (*n* = 3) 1 h p.i. as determined by ROI analysis (Figure 4g). The overall elimination of SeCQDs in CP‐AKI mice was also similar to healthy mice, where 24.8 ± 1.0 %ID was observable at 72 h p.i. (Figure S14c, Supporting Information). The prominent renal uptake of SeCQDs was validated through an ex vivo biodistribution study at 72 h p.i., which showed renal uptake of 31.2 ± 4.2 %ID/g (*n* = 3; Figure 4h). The biodistribution study showed again that SeCQDs evaded any significant accumulation in the liver or spleen, which had uptake values of 2.8 ± 0.5 and 1.8 ± 0.2 %ID/g, respectively.

Due to the ROS scavenging abilities and high renal uptake of SeCQDs, we further investigated if SeCQDs could successfully treat and prevent AKI. AMF is approved by the FDA for the prevention of CP‐AKI and has a therapeutic effect attributed to ROS scavenging. As such, the therapeutic effects of SeCQDs could be compared to the small molecule AMF. In each AKI animal model, the three treatment groups were SeCQDs, AMF, and PBS. Equivalent amounts of AMF and SeCQDs were determined using equimolar selenium and sulfur composition (Table S1, Supporting Information). In the RM‐AKI mice, the doses used were SeCQDs (1 µg in 200 µL), AMF (1µg in 200 µL), and PBS (200 µL). 2 h after initiating RM‐AKI, these agents were injected intravenously. RM‐AKI mice were euthanized 24 h after each agent was administered. The CP‐AKI animal model was monitored by euthanizing mice 24, 48, and 72 h following intraperitoneal injection of cisplatin (Figures S16 and S17, Supporting Information). In the CP‐AKI mice, the doses used were SeCQDs (50 µg in 200 µL), AMF (50 µg in 200 µL), and PBS (200 µL). Agents were administered 1 h (SeCQDs and PBS) or 30 min (AMF) before cisplatin injection.^[^ ^36^ ^]^ To monitor therapeutic efficacy, CP‐AKI mice were euthanized 72 h after cisplatin was administered in each group. Kidney function was assessed similarly in each AKI model, by collecting blood serum samples and kidneys for further analysis.

**Figure 5:**
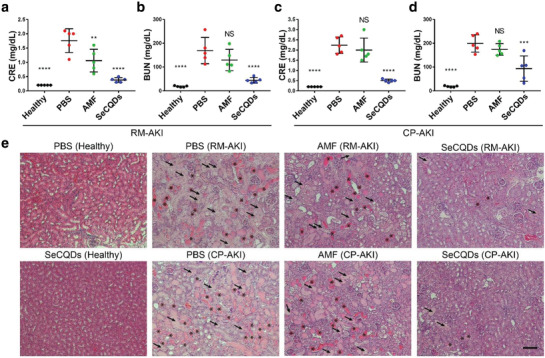
Blood serum biomarker measurements and H&E staining in AKI animal models. a) CRE and b) BUN serum concentrations of groups in the RM‐AKI animal model. c) CRE and d) BUN serum concentrations of groups in the CP‐AKI animal model. e) Histological changes in kidney sections were evaluated after H&E staining. Casts and damaged tubules are indicated with asterisks and arrows, respectively. Scale bar: 100 µm. In (a–d), data represents mean ± s.d. from five independent replicates. *p* values were calculated using one‐way ANOVA with Tukey's honest significant difference post‐hoc test (**p < 0.01, ****p < 0.0001).

Blood serum samples in each group were analyzed for CRE and BUN concentrations (**Figure** **5**a–d; Figures S18 and S19, Supporting Information). CRE and BUN are nitrogenous waste products and accumulate in the blood when kidney function has been impaired. As a result, CRE and BUN are widely used in the clinic to assess kidney function. In RM‐AKI mice, CRE and BUN were dramatically lower for SeCQDs (0.4 ± 0.1 and 44.2 ± 11.7 mg dL^−1^, respectively) compared to PBS (1.8 ± 0.4 and 169.2 ± 55.1 mg dL^−1^, respectively) (*n* = 5). Moreover, SeCQDs successfully prevented CP‐AKI using SeCQDs (CRE: 0.5 ± 0.1, BUN: 93.4 ± 53.3 mg dL^−1^) compared to PBS (CRE: 2.2 ± 0.4, BUN: 199.2 ± 36.4 mg dL^−1^, respectively) (*n* = 5). AMF at an equivalent dose was ineffective in both RM‐AKI (BUN: 129.4 ± 45.0 mg dL^−1^, CRE: 1.06 ± 0.4 mg dL^−1^) and CP‐AKI (BUN: 174 ± 24.3 mg dL^−1^, CRE: 2.0 ± 0.6 mg dL^−1^) mice (*n* = 5).

Kidneys were then analyzed using hematoxylin and eosin (H&E) staining to assess AKI treatment groups for direct histological observations (Figure 5e; Figures S20 and S21, Supporting Information). Indicative of kidney disease, casts are formed in kidneys from the precipitation of denatured proteins. Casts (asterisks) and damaged tubules (arrows) are apparent in PBS or AMF treated mice for both AKI models. In comparison, RM‐AKI and CP‐AKI mice treated with SeCQDs had much less damage. This evidence directly showed that SeCQDs inhibited kidney damage arising from AKI.

**Figure 6:**
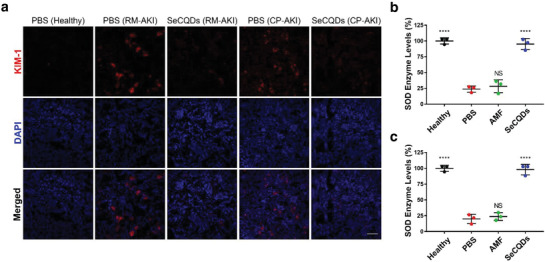
Biomarker and ROS assessment in renal tissue. a) KIM‐1 immunofluorescence staining in AKI and healthy mice. SOD levels in kidney homogenates of b) RM‐AKI and c) CP‐AKI mice. Scale bar: 100 µm. *p* values were calculated using one‐way ANOVA with Tukey's honest significant difference post‐hoc test (****p < 0.0001).

To more directly associate the therapeutic effects of SeCQDs in vivo, kidney tissue sections were resected for confocal imaging. Kidney Injury Molecule‐1 (KIM‐1) is a biomarker that is upregulated on proximal tubules following renal injury.^[^ ^37^ ^]^ As shown in **Figure** **6**a, SeCQDs inhibited KIM‐1 expression compared with PBS controls in both AKI models. Additionally, cleaved caspase‐3, a critical enzyme in initiating apoptosis, was immunofluorescently stained to assess apoptosis in kidney tissue sections.^[^ ^38^ ^]^ In both AKI models, the treatment and prevention of AKI in SeCQDs‐treated mice were supported by decreased apoptosis compared to PBS‐treated mice (Figures S22 and S23, Supporting Information). Kidney homogenates from each treatment group were analyzed for levels of superoxide dismutase (SOD), a critical enzyme responsible for catalyzing the dismutation of superoxide (Figure 6b,c). Excess levels of ROS deplete SOD, which was observed in AKI mice treated with PBS or AMF. However, AKI mice treated with SeCQDs had SOD levels comparable to healthy mice.

**Figure 7:**
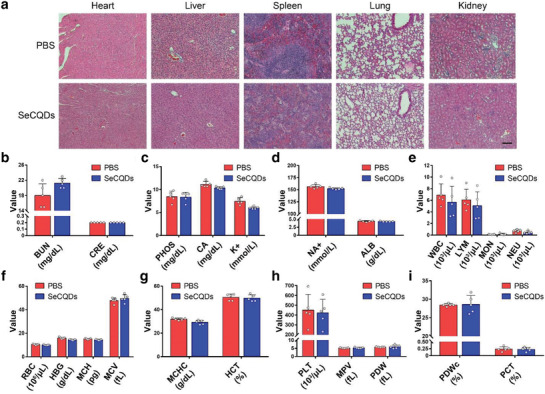
SeCQDs toxicity evaluation. a) H&E staining of major organs (heart, liver, spleen, kidney, lung) for mice injected with SeCQDs or PBS control. Scale bar: 100 µm. Relevant b–d) kidney and e–i) blood parameters were measured to evaluate the toxicity of SeCQDs. In (b–i), data represent mean ± s.d. from five independent replicates.

Any potential toxicity from SeCQDs was assessed by measuring relevant blood parameters and H&E staining of the major organs. Mice were intravenously injected with SeCQDs (50 µg in 200 µL) and then euthanized at 72 h p.i. when blood samples and organs were collected. H&E staining of the major organs shows there are no perceivable histological changes after SeCQDs administration compared to the control (**Figure** **7**a). No renal toxicity from SeCQDs was observed in the kidney profile from blood samples (Figure 7b–d). In addition, the hematology indices of mice injected with SeCQDs were similar to the control (Figure 7e–i). It should be mentioned that only signs of acute toxicity were investigated in this study. The PET imaging studies show that residual SeCQDs remain in the kidneys at 72 h. As such, future studies investigating potential long‐term toxicity are warranted.

## 3 Conclusion

We demonstrated that SeCQDs effectively treat and prevent AKI in murine animal models due to their antioxidant capacity. Experimental results showed effective free radical scavenging of biologically relevant ROS, including O_2_^●−^ and OH^●^, and H_2_O_2_. Due to this, SeCQDs improved cell viability by limiting damage from oxidative stress. PET imaging studies revealed remarkable renal uptake following intravenous administration. Future work will focus on the nano‐bio interactions that favor the renal rather than the RES clearance of SeCQDs. Understanding this has the potential to revolutionize nanomedicine by establishing design criteria that minimize health hazards and govern transport or clearance from living subjects. Researchers will be able to enhance the localization, tolerability, and efficacy of nanomaterials for drug delivery or disease detection. SeCQDs were then shown to be an effective therapeutic option for two clinical cases of AKI, which was confirmed by blood serum measurements, H&E staining, and kidney biomarkers. AMF, at an equivalent dose, was ineffective in the same animal models.

In the clinic, SeCQDs would have advantages and disadvantages compared to other treatment options. SeCQDs could provide robust therapy for treating patients with AKI arising from diverse origins. Additionally, only a low dose of SeCQDs was required to manage the forms of AKI investigated. A primary concern moving forward is the residual amount of SeCQDs remaining in the kidney after administration. While we found no signs of acute toxicity and that it is a small fraction of the injected dose, this requires additional investigation to confirm that no toxicity arises and methods to minimize this uptake. Thus, SeCQDs are a promising nanomaterial option for the clinical management of AKI and might prove propitious in other ROS‐related diseases.

## 4 Experimental Section

##### Materials

L‐selenocystine and Sulfo‐NHS were purchased from Thermo Scientific (Waltham, MA). *N*‐(3‐Dimethylaminopropyl)‐*N*’‐ethylcarbodiiamide hydrochloride (EDC), and Deferoxamine mesylate (DFO) were purchased from Sigma Aldrich (St. Louis, MO). KIM‐1 (TIM‐1) monoclonal antibody was purchased from Bio X Cell (West Lebanon, NH). Anti‐cleaved caspase‐3 antibody conjugated with Alexa Flour 488 was purchased from Novus Biologicals (Centennial, CO). All reagents were used without any purification.

##### Synthesis of SeCQDs

SeCQDs were synthesized using a one‐pot, hydrothermal treatment.^[^ ^17^ ^]^ L‐selenocystine (20 mg) was added to 12 mL of ultrapure water. The pH of the solution was adjusted to ≈9 using 0.5 m NaOH to promote the dissolution of L‐selenocystine. The solution was then stirred at room temperature for 2 h. In an isothermal reactor, the solution was heated at 60 °C for 24 h. The precipitate was removed by centrifugation at 12 000 RPM for 15 min. The supernatant was collected, dialyzed for 24 h using a membrane with a 3.5 kDa MWCO, and then freeze‐dried for further use.

##### DFO Modification of SeCQDs

Following the hydrothermal treatment of SeCQDs and subsequent purification, conjugation with DFO was done for in vivo PET imaging studies. To a SeCQDs synthesis product (yield ≈25%, 5 mg), 2 mg of DFO was added. Subsequently, 46 mg of EDC and 13 mg of Sulfo‐NHS (1:4) was added. To a SeCQDs synthesis product, 46 mg of EDC and 13 mg of Sulfo‐NHS (1:4) was added. The pH of the solution was adjusted to ≈4.5 using 0.5 m HCl. The reaction was carried out for 24 h. Any precipitate was removed by centrifugation at 12 000 RPM for 15 min. The supernatant was collected, dialyzed for 72 h using a membrane with a 10 kDa MWCO, and freeze‐dried for further experiments.

##### Radiolabeling of DFO‐SeCQDs

PET imaging of DFO‐SeCQDs was accomplished through chelation with ^89^Zr. For this 200 µL of SeCQDs was added to 0.5 m HEPES buffer. 1 mCi (37 MBq) of ^89^Zr‐oxalate was added to this solution. The pH of this solution was adjusted to ≈7.5 using 1 m Na_2_CO_3_. Chelation of ^89^Zr occurred by shaking at room temperature for 1 h. A PD‐10 desalting column (GE Healthcare) was used to remove any free ^89^Zr. Radiolabeling yield of ^89^Zr was determined using an autoradiograph of a thin layer chromatography (TLC) plate. After successfully radiolabeling and passing ^89^Zr‐DFO‐SeCQDs through a PD‐10 column, it was incubated in 50% FBS and 20 uL of 1 × 10^−3^ m EDTA was added to 250 uL of ^89^Zr‐DFO‐SeCQDs at 37 °C and shaken at 700 rpm. The radiolabeling stability was evaluated at various time points up to 72 h via TLC.

##### Animal Studies and Ethics

The University of Wisconsin Institution Animal Care and Use Committee (IACUC) approved the protocol for all animal studies conducted. Female CD‐1 Institute of Cancer Research (ICR) mice (4–6 weeks old, Envigo) were used in all animal studies.

##### PET Imaging of ^89^Zr‐DFO‐SeCQDs

Following ^89^Zr labeling of SeCQDs, 80–120 µCi (2.96‐4.44 MBq) of ^89^Zr‐DFO‐SeCQDs was administered intravenously to ICR mice that were either healthy or had AKI induced by either 50% glycerol or cisplatin (*n* = 3). Longitudinal positron emission tomography (PET) scans were obtained using an Inveon microPET/CT rodent model scanner (Siemens Healthineers, Germany). At the time point of interest, mice were anesthetized with isoflurane (2% in oxygen) and placed on the bed of the PET scanner. During the initial three time‐points (5 min, 1 h, 4 h) p.i., 40 million coincidence events were collected whereas 30 million were collected for the remaining. PET data was reconstructed by 3D ordered‐subset expectation maximization followed by maximum a posteriori reconstruction (OSEM3D/MAP) and decay corrected using the Inveon Research Workplace software (Siemens Healthineers, Germany). Region‐of‐interests were drawn at each time‐point to quantify the biodistribution in each tissue of interest (blood, liver, spleen, kidney, bone). After the PET scan at the terminal time‐point, the mice were euthanized, and the organs were collected to quantify biodistribution ex vivo using a gamma counter (Perkin Elmer, USA). SeCQDs uptake in tissue was determined as a percentage of the injected dose per gram of tissue (%ID/g).

##### ABTS Free Radical Scavenging Using SeCQDs

The free radical scavenging capacity of SeCQDs was evaluated using the ABTS decolorization assay. ABTS radical cations (ABTS^+●^) were formed by reacting 7mm ABTS with 2.45 mm potassium persulfate in ultrapure water for 12 h before use. The resulting solution was then diluted using ultrapure water until an absorbance value of 0.7 ± 0.02 was measured at 734 nm. Using a 96‐well plate, 180 µL of diluted ABTS^+●^ solution was added with the corresponding concentration of SeCQDs (0, 1, 10, 25 µg mL^−1^). The ABTS scavenging efficiency was determined by measuring the absorbance at 734 nm and calculated by: Scavenging efficiency = (*A*_B_ − *A*_E_)/*A*_B_*100, where *A*_B_ is the absorbance of the blank sample and *A*_E_ is the absorbance of the SeCQDs sample.

##### Superoxide Anion Scavenging Using SeCQDs

The superoxide anion scavenging capacity of SeCQDs was evaluated using a SOD assay kit (Dojindo Molecular Technologies, Inc, USA). Experiments were conducted using the protocol provided by the manufacturer.

##### Hydroxyl Radical Scavenging Using SeCQDs

The hydroxyl radical scavenging capacity of SeCQDs was evaluated using a hydroxyl radical antioxidant capacity (HORAC) assay kit (Cell Biolabs, Inc., USA). Experiments were conducted following the protocol provided by the manufacturer.

##### Viability of Cells Protected by SeCQDs from Oxidative Stress

Human embryonic kidney 293 (HEK293) was cultured in Dulbecco's Modified Eagle Medium (DMEM) (1% penicillin/streptomycin and 10% fetal bovine serum) at 37 °C under 5% CO_2_. Using a 96 well plate, HEK293 cells were seeded at a concentration of 1 × 10^4^ cells per well and incubated for 24 h. Next, SeCQDs dispersed in the cell culture medium at various concentrations (0, 25, 50, 100 µg mL^−1^) were added to the wells and incubated for 30 min. H_2_O_2_ was then added to the cells to a final concentration of 250 µm and incubated for 24 h. Cells without any treatment, treated with only SeCQDs, or treated with only H_2_O_2_ were used as controls. The standard MTT assay was used to measure cell viability.

##### Confocal Imaging of ROS Production and Mitochondria

Human embryonic kidney 293 (HEK293) were cultured in Dulbecco's Modified Eagle Medium (DMEM) (1% penicillin/streptomycin and 10% fetal bovine serum) at 37 °C under 5% CO_2_. Staining for confocal imaging was performed as described in the protocol supplied by the manufacture. Briefly, after treating cells with a final concentration of 250 µm H_2_O_2_ and SeCQDs (100 µg mL^−1^) for 24 h, CellROX Deep Red Reagent (Invitrogen) or Mitrotracker green (Invitrogen) was added to the cells and incubated for 30 min. Cells were then washed three times with PBS and used directly for confocal imaging.

##### Rhabdomyolysis‐induced AKI Model

ICR mice were deprived of water but were given food ad libitum for 15 h. Following the water restriction, 8 mL kg^−1^ of 50% glycerol was administered intramuscularly into both hind limbs of mice equally. The mice were then given access to water and food. After 24 h, the mice were euthanized before collecting blood and kidney samples. For treatment, SeCQDs were injected intravenously 2 h after glycerol injection.

##### Cisplatin‐Induced AKI model

ICR mice were intraperitoneally injected with cisplatin (20 mg kg^−1^) to induce kidney injury. The model development was monitored by sacrificing mice at 1, 2, or 3 days p.i. of cisplatin. Kidneys were collected and blood samples analyzed at each time point.

##### Treatment of AKI Mice

Five groups were used for studies using mice with glycerol‐ and cisplatin‐induced AKI: group 1 was healthy mice treated with 1 × PBS (*n* ≥ 5); group 2 was healthy mice (*n* ≥ 5) treated with SeCQDs (50 µg in 200 µL PBS), group 3 was AKI mice treated with 1 × PBS (*n* ≥ 5), group 4 was positive control group that AKI mice treated with 1 or 50 µg of AMF in 200 µL of PBS for RM‐AKI and CPPD‐AKI mice, respectively (*n* ≥ 5); group 5 was AKI mice treated with 1 or 50 µg of SeCQDs in 200 µL of PBS for RM‐AKI or CPPD‐AKI, respectively (*n* ≥ 5). Treatment groups were intravenously injected into rhabdomyolysis‐AKI mice 2 h after intramuscular injection of 50% glycerol. In mice with cisplatin‐induced AKI, treatment groups were intravenously injected 30 min (AMF only) or 1 h (all others) before intraparietal injection of cisplatin. RM‐AKI mice were euthanized 24 h p.i. of the treatment group and compared to healthy mice to evaluate kidney function. CP‐AKI mice were euthanized 72 h p.i. of cisplatin and compared to healthy mice to evaluate kidney function.

##### Kidney Function Test

After euthanizing the mice, blood samples were collected in lithium heparin tubes (BD Biosciences, USA). The blood serum was collected after centrifuging the blood samples at 2000 g for 15 min at 4 °C. The Clinical Pathology Laboratory performed analysis of serum BUN and CRE concentrations at the University of Wisconsin Veterinary Medical Teaching Hospital.

##### Haemotoxylin and Eosin (H&E) Staining of Kidney Sections

In the AKI model and time‐point of interest, mice were euthanized and the kidneys collected and fixed with paraformaldehyde (4% in PBS). The University of Wisconsin Carbon Cancer Center embedded the samples in paraffin wax, sectioned, and then H&E stained them.

##### Analysis of Renal Tissues after Treatment

Kidneys from AKI groups were frozen and stored at −80 °C. Kidney homogenates were prepared as required for the SOD assay.

##### Confocal Imaging of KIM‐1 Expression in Kidney Tissue

Kidneys were resected from mice and then stored at −80 °C in optimum cutting temperature (O.C.T.) compound. Tissues were sectioned (≈5 µm) by the Experimental Pathology Laboratory at the University of Wisconsin‐Madison. For immunofluorescence staining, kidney section slides were allowed to dry for 15 min at room temperature. The slides were then fixed with 4% paraformaldehyde for 10 min, washed with PBS twice for 5 min each, permeabilized with 0.2% Triton X‐100 in PBS for 15 min, and then washed three times with PBS for 5 min each. Nonspecific binding was blocked using 5% horse serum/0.3% Triton X‐100 in PBS for 1 h. Kidney sections were then incubated with 10 µg mL^−1^ of rat anti‐mouse KIM‐1 antibody or 3.75 ug mL^−1^ anti‐cleaved caspase 3‐Alexa Fluor 488 overnight at 4 °C. The kidney slides were then washed three times with 0.2% Triton and then three times with PBS for 10 min each. The secondary antibody, Cy3‐labeled donkey anti‐rat IgG, was then incubated with the kidney sections for 1 h at room temperature. No secondary antibody was used for cleaved caspase‐3 staining and the slides were directly mounted with Hard‐set Mounting Medium containing DAPI. After additional washing with 0.2% Triton and PBS, the slides were mounted with Hard‐set Mounting Medium containing DAPI.

##### SeCQDs Toxicity Evaluation

ICR mice were intravenously injected with either SeCQDs (50 µg per mouse in 200 uL) or PBS (200 uL) as controls. The mice were euthanized 72 h p.i. when blood samples and major organs collected. Complete blood panel screening and kidney profiling were then performed. The measured blood parameters were red blood cells (RBC), hemoglobin (HGB), mean corpuscular hemoglobin (MCH), mean corpuscular volume (MCV), white blood cells (WBC), lymphocyte (LYM), monocytes (MON), neutrophils (NEU), mean corpuscular hemoglobin concentration (MCHC), hematocrit level (HCT), platelets (PLT), mean platelet volume (MPV), platelet distribution width (PDW), and procalcitonin (PCT). The relevant kidney parameters measured using the Abaxis, Inc (Union City, CA, USA) VS2 Kidney Profile Plus were blood urea nitrogen (BUN), creatinine (CRE), phosphorous (PHOS), calcium (CA), potassium (K+), sodium (NA+), and albumin (ALB). In addition, the collected heart, liver, spleen, lung, and kidney from each group were sectioned and any histological changes were determined after H&E staining.

##### Instrumentation

UV–vis spectra were measured on an Agilent Cary 60 spectrophotometer. Zeta potential measurements were performed on a Nano‐Zetesizer (Malvern Instruments Ltd). TEM imaging was performed using an FEI Technai T‐12 microscope (Hillsboro, Oregon). XPS measurements were performed on a Thermo Scientific K‐alpha XPS. FTIR measurements were performed using a Bruker Equinox 55 (Billerica, MA). H&E staining was observed on a Nikon Eclipse Ti‐U inverted optical microscope (Japan). Confocal images were taken using a Nikon A1RS confocal microscope (Japan).

##### Statistical Analysis

Quantitative data were expressed as mean ± s.d, *: *p* < 0.05; **: *p* < 0.01; ***: *p* < 0.001; ****: *p* < 0.0001.

## Conflict of Interest

The authors declare no conflict of interest.

## Author Contributions

Z.T.R. and W.B.C. conceived the idea and designed the study. Z.T.R. and T.W.S. conducted the experiments with the help of D.W.J., W.Y.C., and C.A.F. Z.Y.Z. performed the AFM imaging. T.E.B. and J.W.E. produced the radioisotopes. X.D.W. contributed through discussion. D.W.J., P.H., and W.B.C. supervised the research. All authors assisted in preparing the manuscript.

## Supporting information

Supporting Information
