# Supplementary material for: Selenium‐Doped Carbon Quantum Dots Act as Broad‐Spectrum Antioxidants for Acute Kidney Injury Management
Source: Adv Sci (Weinh). 2020 Apr 29;7(12):2000420. doi: 10.1002/advs.202000420 (PMC7312409; doi:10.1002/advs.202000420)
Supplement: Supplementary file 1 — Supporting Information [file ADVS-7-2000420-s001.pdf]

Copyright WILEY-VCH Verlag GmbH & Co. KGaA, 69469 Weinheim, Germany, 2020.

Supporting Information

Selenium-Doped Carbon Quantum Dots Act as Broad-Spectrum Antioxidants for Acute Kidney Injury Management

Zachary T. Rosenkrans, Tuanwei Sun, Dawei Jiang, Weiyu Chen, Todd E. Barnhart, Ziyi Zhang, Carolina A. Ferreira, Xudong Wang, Jonathan W. Engle, Peng Huang*, and Weibo Cai**

Z. T. Rosenkrans, Prof. W. Cai
Department of Pharmaceutical Sciences, University of Wisconsin-Madison, Madison, WI, 53705, USA
E-mail: wcai@uwhealth.org

Dr. T. Sun, Dr. D. Jiang, Prof. P. Huang
Marshall Laboratory of Biomedical Engineering, International Cancer Center, Laboratory of Evolutionary Theranostics, School of Biomedical Engineering, Shenzhen University Health Science Center, Shenzhen 518060, China
E-mail: peng.huang@szu.edu.cn

Dr. T. Sun, Dr. D. Jiang, Dr. W. Chen, Dr. T. E. Barnhart, Dr. C. A. Ferreira, Prof. J. W. Engle, Prof. W. Cai
Departments of Radiology and Medical Physics, University of Wisconsin-Madison, Madison, WI, 53705, USA
E-mail: djiang29@wisc.edu

Dr. Z. Zhang, Prof. X. Wang
Department of Materials Science and Engineering, University of Wisconsin-Madison, Madison, WI, 53705, USA

Keywords: Selenium, Carbon Quantum Dots, Acute Kidney Injury, Cisplatin, Rhabdomyolysis, positron emission tomography, nanomedicine

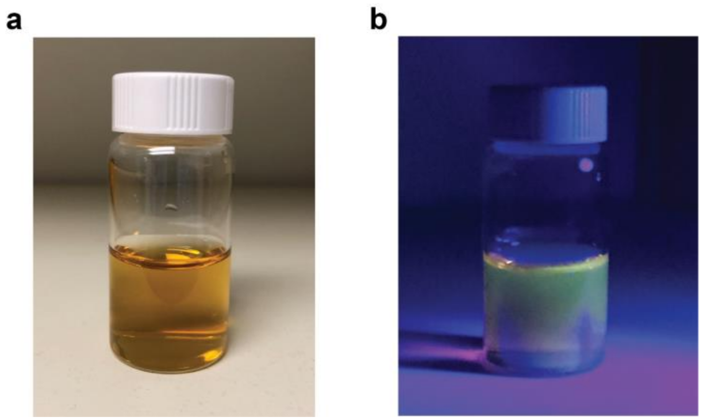

Figure S1. Pictures of SeCQDs under (**a**) normal and (**b**) UV/Blue light.

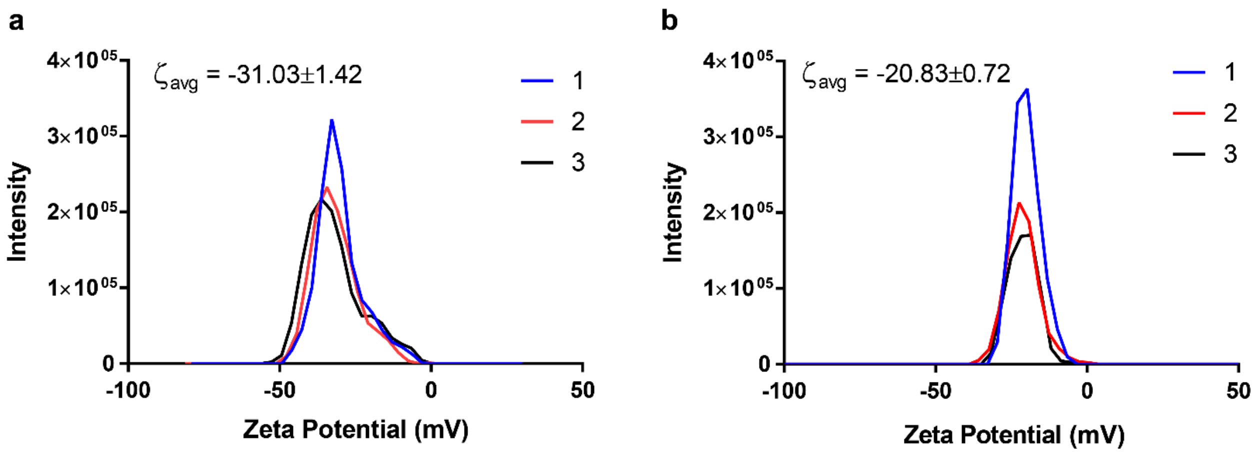

Figure S2. Zeta potential of (a) SeCQDs and (b) DFO-SeCQDs

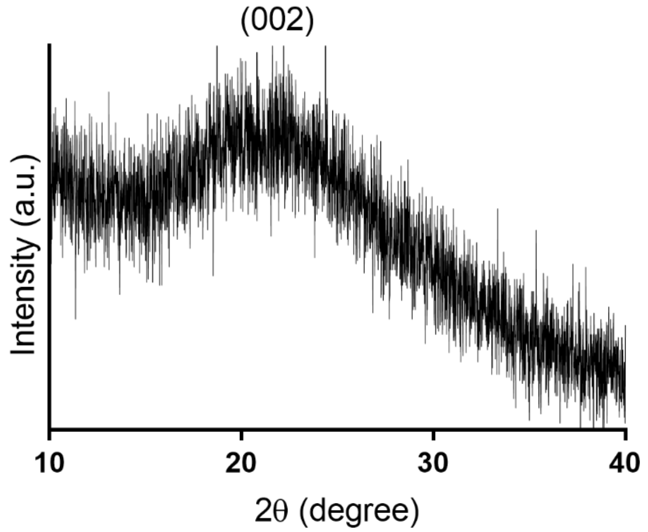

Figure S3. XRD spectra of SeCQDs.

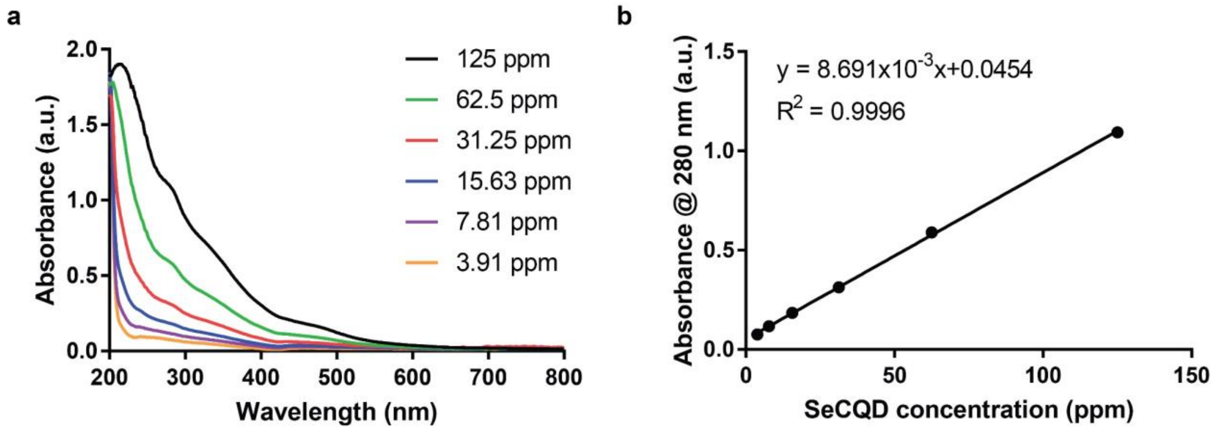

Figure S4. UV-Vis Spectrum of SeCQDs. **a** UV-Vis measurements at various concentrations (**b**) Correlation used to relate absorbance and concentration at 280 nm.

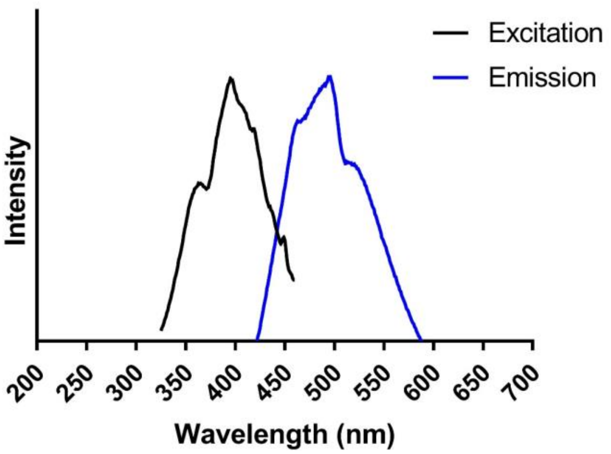

Figure S5. Fluorescence spectra of SeCQDs.

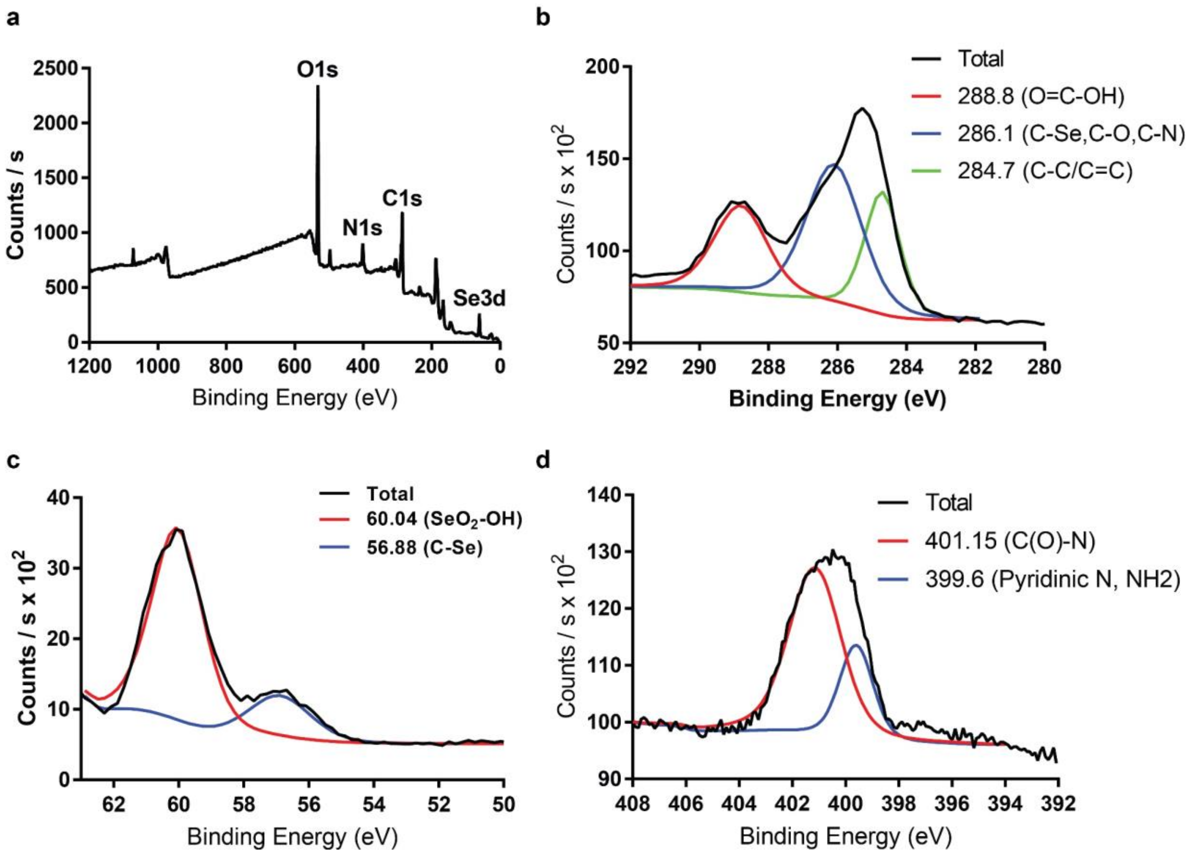

Figure S6. XPS studies of oxidized SeCQDs using H_2O_2. **a** Survey spectra of oxidized SeCQDs. High resolution spectra of (**b**) carbon, (**c**) selenium, and (**d**) nitrogen.

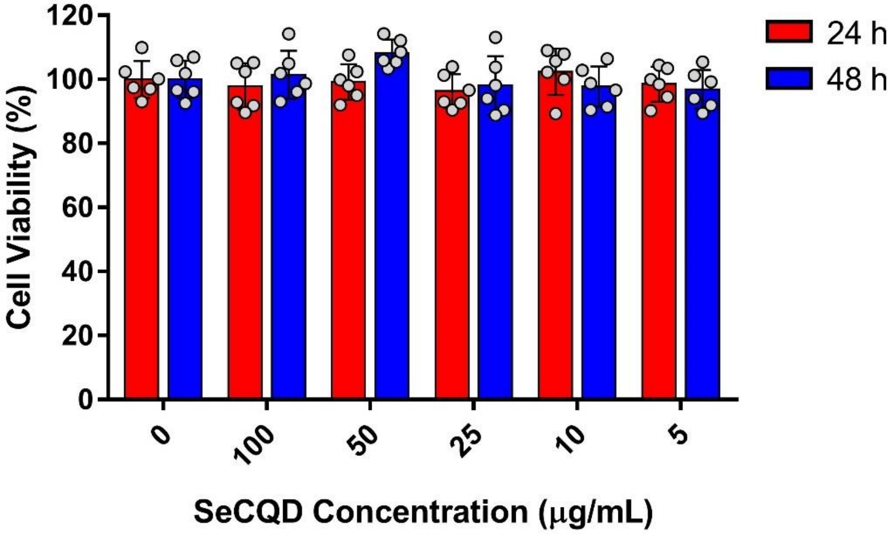

Figure S7. MTT assay of SeCQDs using HEK293 cells for toxicity evaluation.

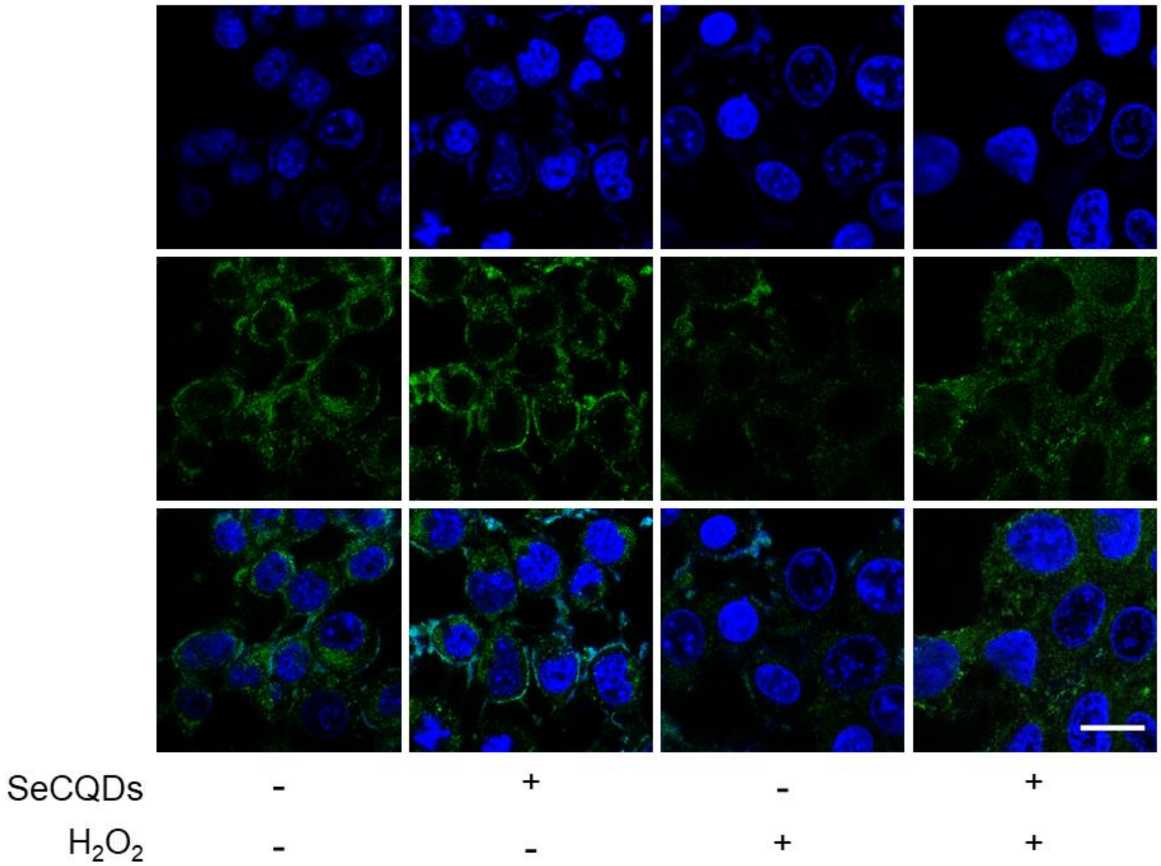

Figure S8. Representative mitochondrial staining of HEK-293 cells treated with H_2O_2 and SeCQDs. Scale bar 20 um.

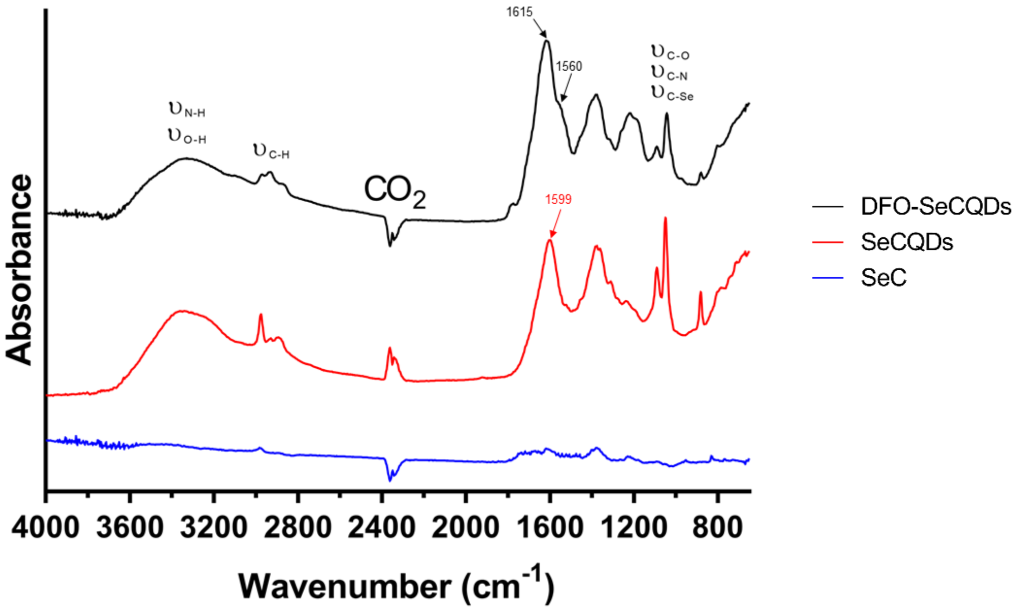

Figure S9. FTIR spectra for DFO-SeCQDs, SeCQDs, and SeC.

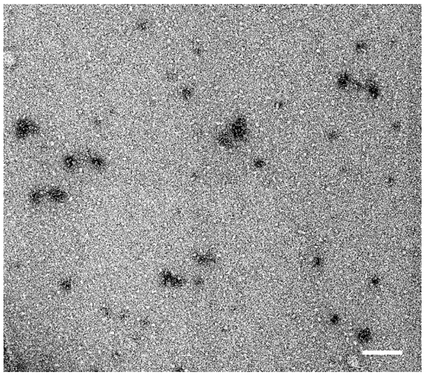

Figure S10. TEM image of DFO-SeCQDs. Scale bar 200 nm.

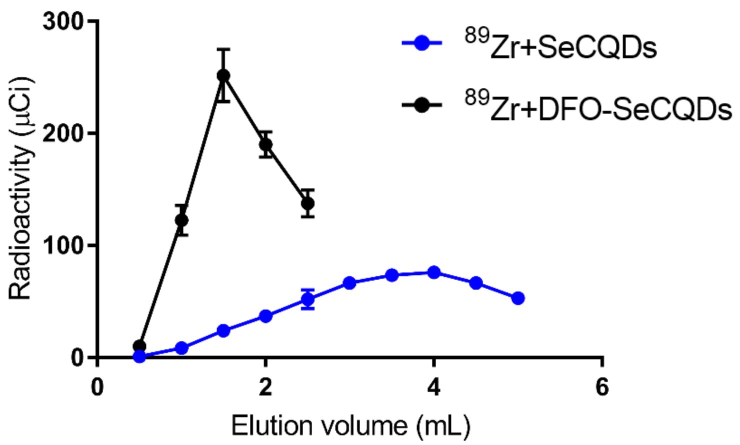

Figure S11. Comparison of Zr-89 radioactivity eluted from PD-10 columns when labeled with SeCQDs (^{89}Zr+SeCQDS) or DFO-SeCQDs (^{89}Zr+DFO-SeCQDs).

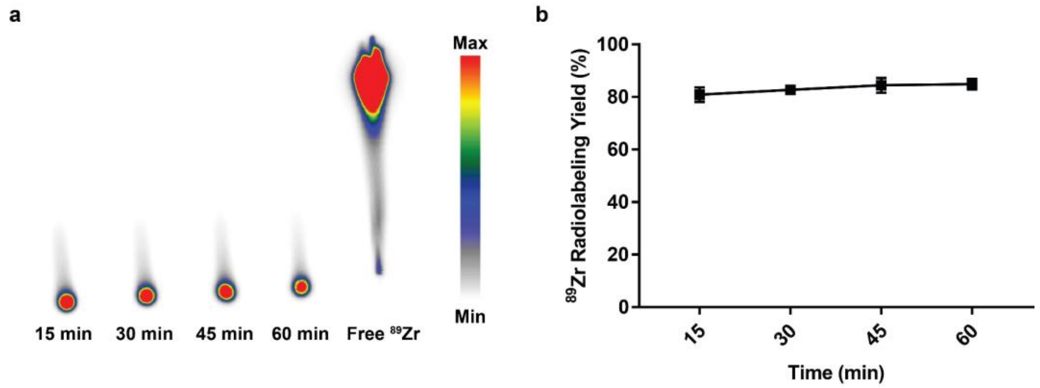

Figure S12. Radiolabeling stability of SeCQDs. **a** representative TLC radiolabeling image. **b** TLC radiolabeling stability over a 1 h period (n=3).

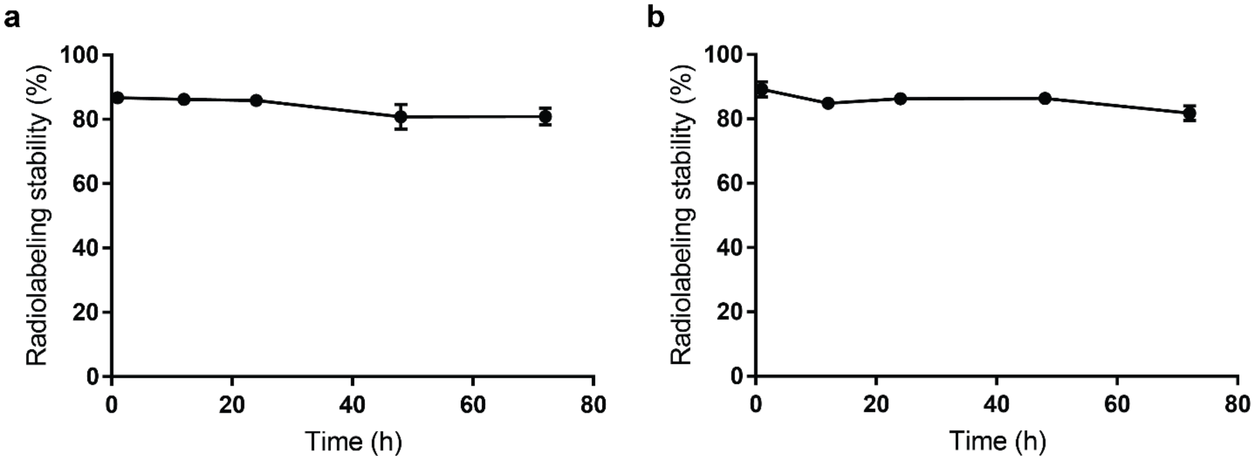

Figure S13. Radiolabeling stability of ^{89}Zr-DFO-SeCQDs incubated in (a) 50% FBS and (b) PBS + EDTA at 37°C.

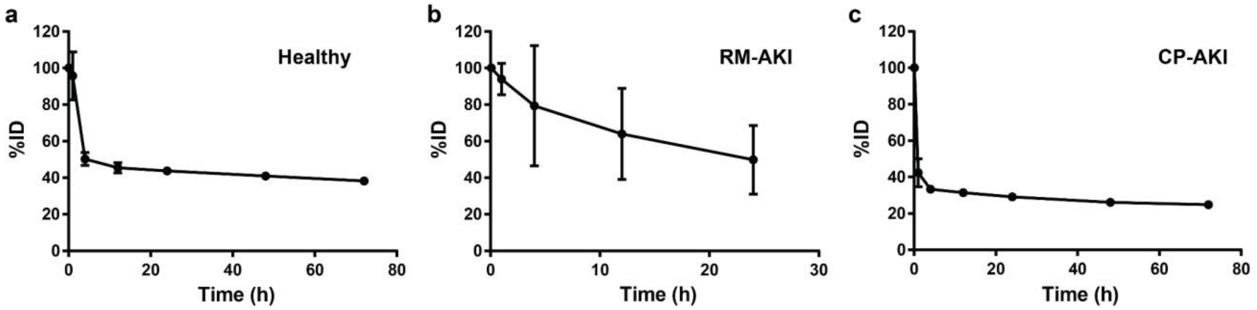

Figure S14. The percent injected dose (%ID) was quantified using ROI analysis in (**a**) healthy, (**b**) RM-AKI, and (**c**) CP-AKI mice to evaluate the overall retention of SeCQDs in mice at different time points.

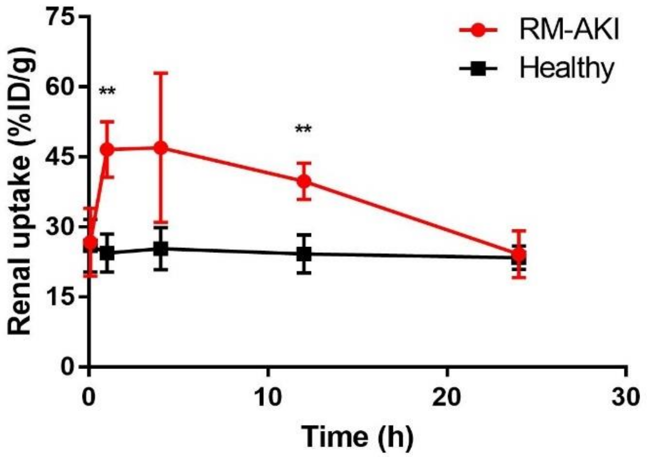

Figure S15. Comparison of renal uptake of ^{89}Zr-DFO-SeCQDs in healthy and RM-AKI mice. P-values were calculated by two-tailed Student's (**P<0.01).

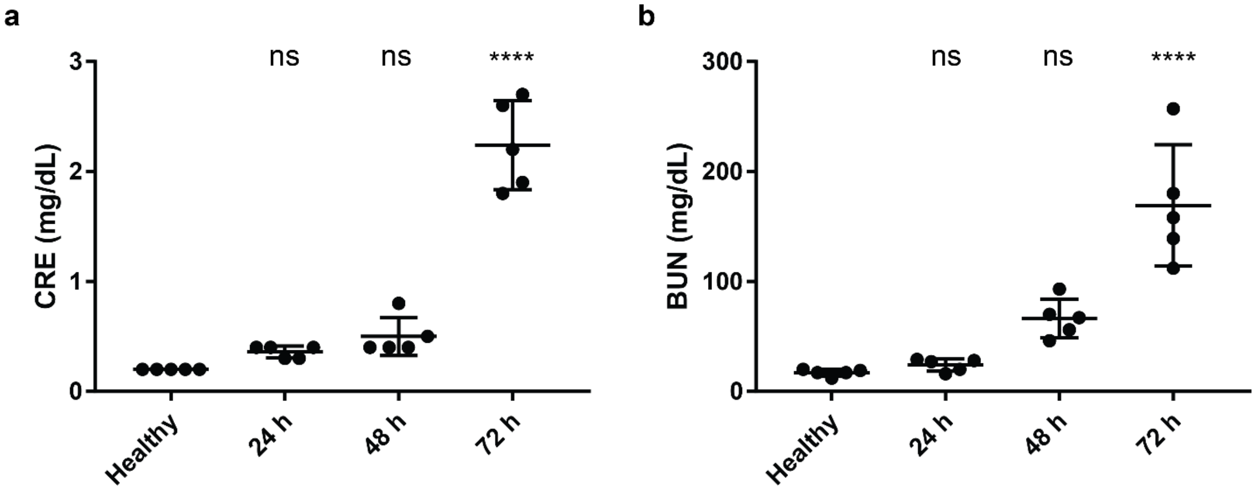

Figure S16. CP-AKI model establishment. Development of the CP-AKI animal model was monitored over 72 h by measuring (**a**) CRE and (**b**) BUN serum concentrations. P values were calculated using one-way ANOVA with Tukey's honest significant difference post-hoc test (****P<0.0001, ns: not significant).

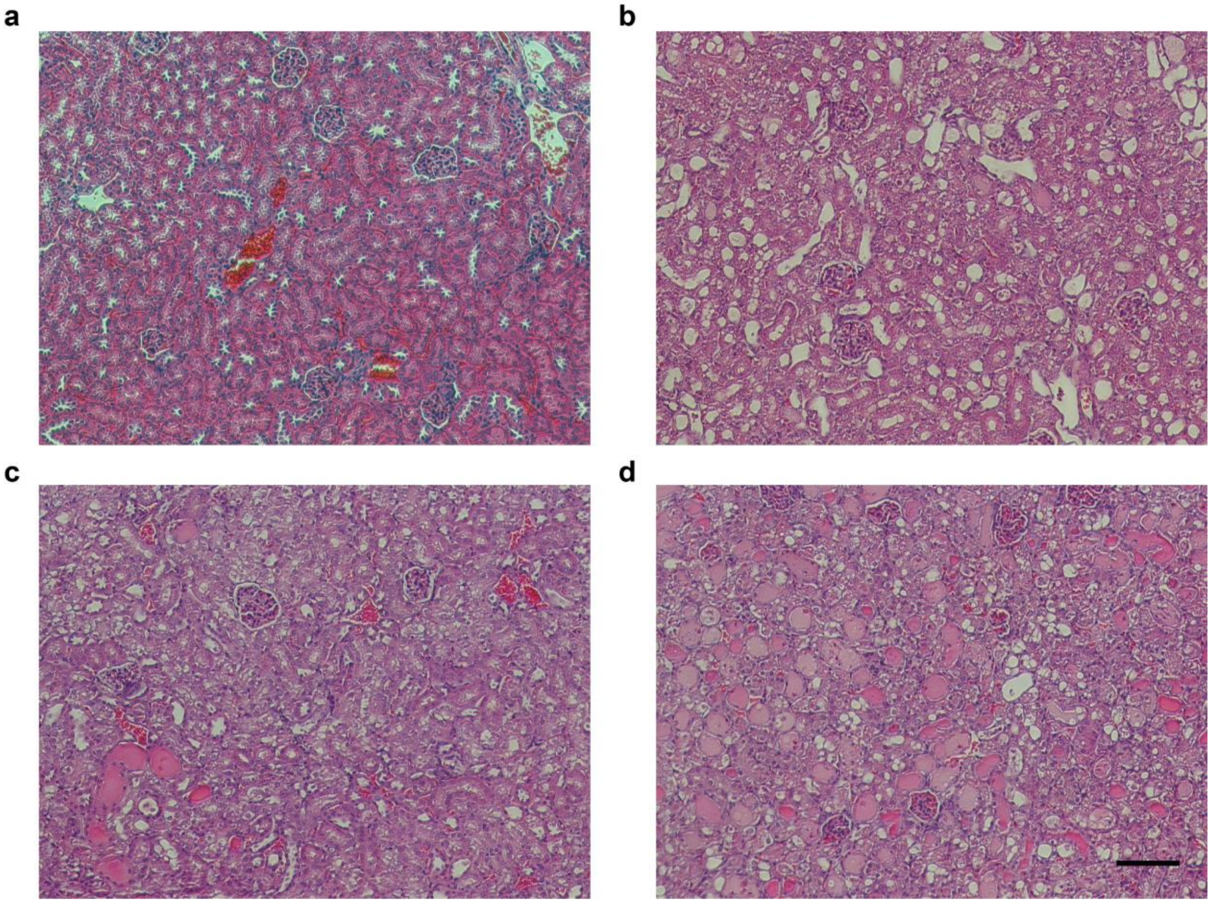

Figure S17. H&E staining of CP-AKI model establishment. The histological changes in kidney tissues was monitored by H&E staining as the CP-AKI model progressed from (**a**) healthy mice to (**b**) 24 h, (**c**) 48 h, and (**d**) 72 h after administering cisplatin. Scale bar: 100 μm.

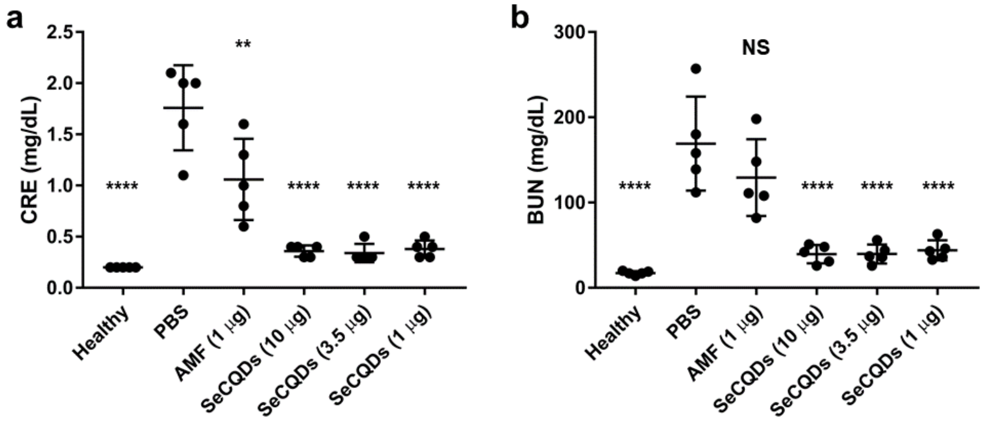

Figure S18. Summary of (**a**) CRE and (**b**) BUN for all groups and doses of RM-AKI mice. P values were calculated using one-way ANOVA with Tukey's honest significant difference post-hoc test (**P<0.01, ****P<0.0001, NS: not significant).

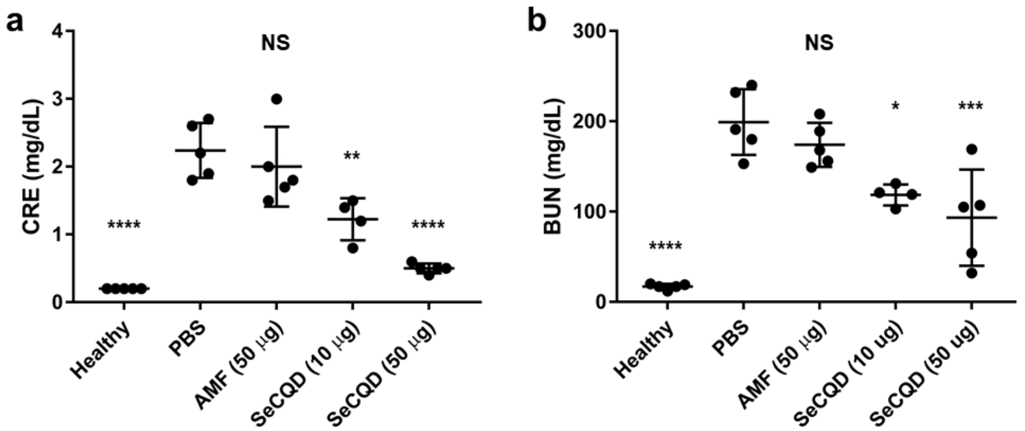

Figure S19. Summary of (**a**) CRE and (**b**) BUN for all groups and doses of CP-AKI mice. P values were calculated using one-way ANOVA with Tukey's honest significant difference post-hoc test (*P<0.05., **P<0.01, ***P<0.001, ****P<0.0001, NS: not significant).

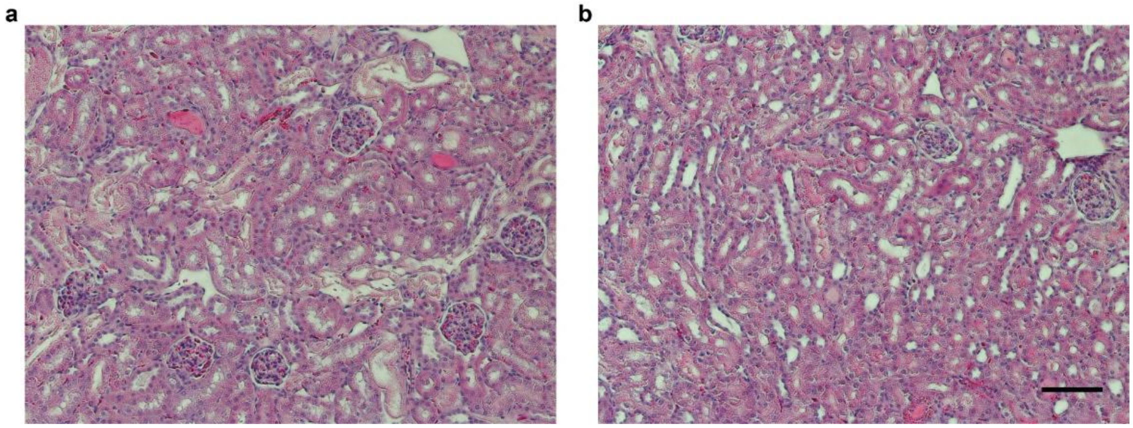

Figure S20. Representative H&E staining of kidney sections from RM-AKI mice treated with (**a**) 3.5 μg or (**b**) 10 μg of SeCQDs. Scale bar: 100 μm.

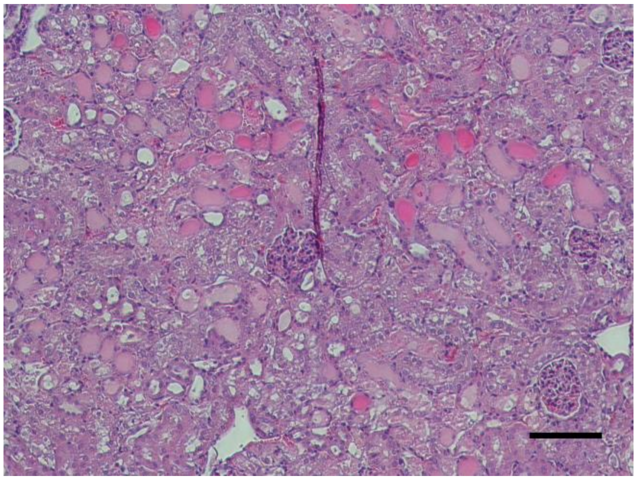

Figure S21. Representative H&E staining of kidney sections from CP-AKI mice treated with 10 μg of SeCQDs. Scale bar: 100 μm.

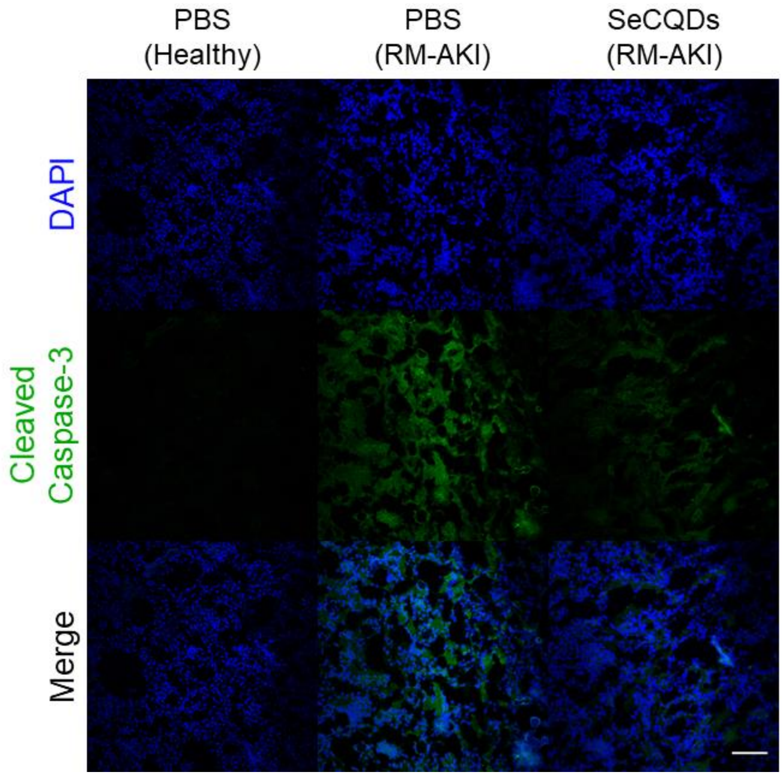

Figure S22. Cleaved caspase-3 staining for RM-AKI mice. Scale bar: 100 μm.

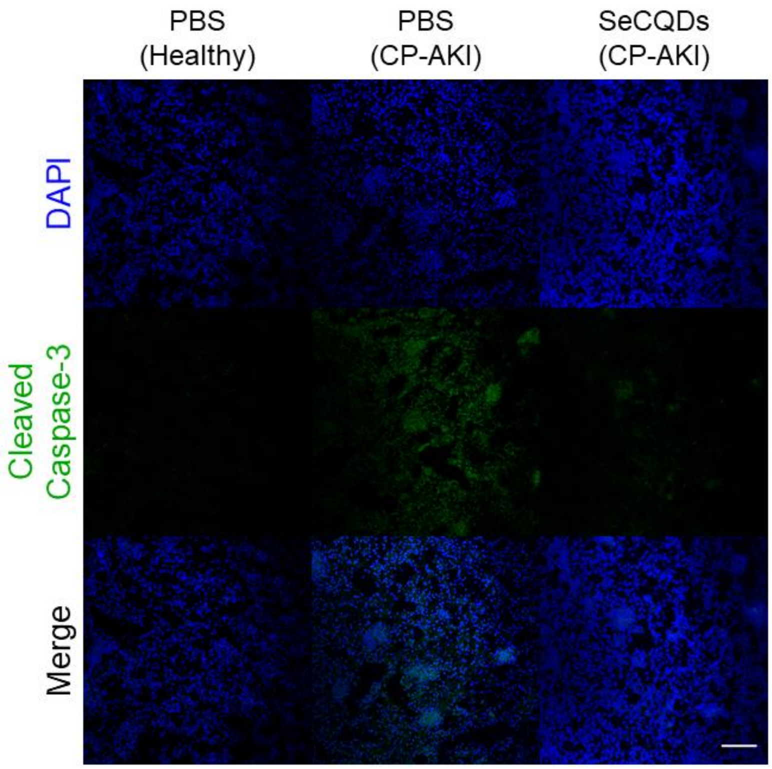

Figure S23. Cleaved caspase-3 staining for CP-AKI mice. Scale bar: 100 µm.

Table S1. Calculations to determine equivalent doses of SeCQDs and AMF. SeCQDs were found to be 29% Se (by weight) using XPS.

Sample	Model	Dose (ug)	Dose of Se (ug)	umol of Se	umol of S	AMF (ug)
SeCQD	RM-AKI	1	0.29	0.004	-	-
SeCQD	CP-AKI	50	14.5	0.184	-	-
AMF	RM-AKI	-	-	-	0.004	1.0
AMF	CP-AKI	-	-	-	0.184	49.3
